# The language of discovery

**DOI:** 10.5210/disco.v6i0.3634

**Published:** 2011-06-17

**Authors:** Wiley Souba

**Affiliations:** 1 Office of the Dean, Dartmouth Medical School and Vice-President for the Health Affairs at Dartmouth College, 1 Rope Ferry, Hanover, NH 03755United States

## Abstract

Discovery, as a public attribution, and discovering, the act of conducting research, are experiences that entail “languaging” the unknown. This distinguishing property of language ‐ its ability to bring forth, out of the unspoken realm, new knowledge, original ideas, and novel thinking – is essential to the discovery process. In sharing their ideas and views, scientists create co‐negotiated linguistic distinctions that prompt the revision of established mental maps and the adoption of new ones. While scientific mastery entails command of the conversational domain unique to a specific discipline, there is an emerging conversational domain that must be mastered that goes beyond the language unique to any particular specialty. Mastery of this new conversational domain gives researchers access to their hidden mental maps that limit their ways of thinking about and doing science. The most effective scientists use language to recontextualize their approach to problem‐solving, which triggers new insights (previously unavailable) that result in new discoveries. While language is not a replacement for intuition and other means of knowing, when we try to understand what’s outside of language we have to use language to do so.

## Introduction 

On February 28, 1953, James Watson and Francis Crick walked into the Eagle pub in Cambridge, England to have lunch. Crick promptly announced to those present in the tavern: “We have found the secret to life.” He was not kidding. Two months later, in their landmark paper in Nature(1), they wrote, “It has not escaped our notice that the specific pairing we have postulated immediately suggests a possible copying mechanism for the genetic material.” Watson and Crick had discovered the double-helical structure of DNA, cracking the code of genetic instructions for all life on earth. Their breakthrough discovery transformed the world of science, ushered in the age of molecular biology, and opened up vast new possibilities for the application of nucleic acid research.

We can empirically assume that nucleic acids have existed since life on earth began several billion years ago. However, prior to Watson and Crick’s findings, what we know today as the DNA double helix existed only in the unspoken and unaware domain of language. When they wrote, “This (DNA) structure has two helical chains each coiled round the same axis.... Both chains follow right handed helices [and] the two chains run in opposite directions…. The bases are on the inside of the helix and the phosphates on the outside”, the unknown became known. 

The significance of “revealing” a double helix that “unzipped” was not in labeling something that was always already there, but in making available new knowledge that allowed scientists to relate to and engage with the world more meaningfully. What was unknown was “languaged,” emancipated for participation in the world. When what is unknown is made accessible to human beings, new possibilities for applying that knowledge are created. Watson and Crick’s discovery paved the way for sequencing the human genome and the birth of new knowledge domains, to include bioengineering, molecular genetics, bioinformatics, and personalized medicine. 

Watson and Crick didn’t create DNA but they did create access to it. In making the unknown known, it was made available for human use, a process that involves language. Language allows us to turn events into “talkable” objects, making them accessible in the sense that we can name them, ponder them, contest them, and “get our hands” on them. Those events we cannot talk about, we cannot contend with. For example, dark energy, the primary component of the universe, has been labeled, but to date remains inadequately “languaged” to make it accessible for human understanding and use.

What was DNA before it was named? What was gravity before it was named? Certainly these natural phenomena existed in the world but they were “unlanguaged.” Human beings can only inhabit and make meaning of the world that has been distinguished in language by human thinking. Are there “things” that exist without language? How did our distant ancestors perceive clouds before there was language to make sense of them? Did they possess some sort of mind stuff that was the equivalent of “white” or “fluffy” or “wispy”? Pinker uses the term mentalese to refer to concepts and propositions that are represented in the mind without words (2). Certainly there are “things” that are independent of language or “beyond” the facility of language to symbolize them, but they remain elusive because we have no language with which to talk about them. We can only deal with – understand, solve, evaluate – things we can talk about.

The purpose of this paper is to review the role of language in enabling the process of discovery. Both the “unveiling” property of language as well as its imprecision and limitations are examined. While scientific mastery entails command of the conversational domain unique to a specific discipline, there is an emerging conversational domain that must be mastered that goes beyond the language unique to any particular specialty. Mastery of this new conversational domain gives researchers access to their hidden mental maps and frames of reference that limit their ways of thinking about and conducting science, thereby creating new possibilities for furthering the discovery process. 

## Language is Central in Constituting, Constructing, and Creating the Human World 

Conventional thinking understands language as symbolic: the world is presented to human perception in a certain way, and the task of language is to re-present things in words the way they are in the world (3-5). Inside of this deeply engrained worldview, the world is the way it is before language – words are merely labels or symbols for what is always already there. This view of language as a symbolic, referential exchange system is not wrong but it is limited. Rather, language is first and foremost “constitutive” of the human world and thus, is intricately linked to who and how we are (6-10). Constitutive (con´stitutive, emphasis on first syllable) as used here means having the power to establish, create, or make a thing what it is. This designation is differentiated from the other meaning of constitutive (consti´tutive, emphasis on second syllable), which denotes a constant rate, e.g., constitutive gene expression.

The constitutive view of language says the world is not the way it is before language. Rather, objects, people, situations, and experiences in our lives come to be what they are for us in and through language. Before language, a “thing” – a mountain, a molecule, a mosquito - is only an occurring, a happening, an event. Moneta (11) writes:

In the most general sense, the experience of the object which is prior to our predicating anything about it is the ‘discourse’ that the object makes of itself through the particular mode in which it presents itself.

The role of “languaging” (speaking), explains Anton (3), is not just about the social exchange of preexisting ideas, i.e., encoding and decoding; speech also enables the emergence, formation, and concrete accomplishment of thought. Human meaning is not givena priori and then re-“called” in words; rather, language itself, at work in our everyday conversations, adds the meanings, opening up to us a distinctively *human* world.  

It is useful to point out that language incorporates much more than the spoken word. It includes self-talk (thought) and the unspoken. I argue here that language is always involved (at least to some extent) in the process of sense-making in discovery. Much of discovery begins with visual inputs, which activate neural networks involved in perception. Interpretation of these inputs is brought into consciousness firstly by way of thought. Hyde (12) elaborates:

The development of understanding by interpretation occurs in a “working out” of linguistic possibilities whereby understanding reveals itself in the foresight and the fore-conception of the culture’s members…. [T]he making-known function of discourse occurs initially in a person’s thinking…. Discourse brings the word to mind such that [what is being interpreted] can be thought and eventually made-known to others through communication practices….

A stunning example of the role of language in creating the world is found in Helen Keller's autobiographical account of her early childhood. Unable to see or hear from age one, she was both worldless and wordless, completely unable to discover life. She writes, “Before my teacher came to me, I did not know that I am. I lived in a world that was no-world. My inner life, then, was a blank without past, present, or future….” (13). Later, Keller recounts an incident with her teacher that resulted in her first experience of discovering the world:

As the cool stream gushed over one hand she spelled into the other the word water, first slowly, then rapidly. I stood still, my whole attention fixed upon the motions of her fingers. Suddenly I felt a misty consciousness as of something forgotten, a thrill of returning thought; and somehow the mystery of language was revealed to me. I knew then that “w-a-t-e-r” meant the wonderful cool something that was flowing over my hand. That living word awakened my soul, gave it light, hope, joy, set it free! (14).

The occurring world - the only world we know - is a world that is sometimes constituted in language, and when it is not, it is at least colored and shaped by language, and is invariably accessible through language (10). Each of us is looking at the world through the lenses of our accumulated contexts and perspectives, which significantly arise in, reside in, and are continuously molded by language. Heidegger reminds us that “we do not say what we see, but rather the reverse, we see what one says about things” (6). He adds, “Only where the word for the thing has been found is the thing a thing…. Accordingly we must stress as follows: no thing is where the word, that is, the name, is lacking” (7).

The scientist who fails to keep in mind that reality is largely a language construct may presume that the world “occurs” for others the way it “occurs” for him. As noted by Hyde (15), this recognition of the researcher's inevitable presence in his or her own observations has particular relevance for the study of communication:

That is, my relationship with the object of my research is circular and mutually determinative: my interpretive context (i.e., my theoretical, methodological, and epistemological presuppositions) shapes my interaction with my research object, and that interaction recursively reshapes my interpretive context. The reshaped context, in turn, more fully determines my further observations.

Take the term DNA. There is no particular reason why “DNA” should refer to a double helix molecular structure that forms the basis of genetic inheritance. DNA was around long before humans developed the use of language, but there was no possibility of “DNA” until its meaning was brought into being by human thought. In other words, things come to be the things they are in language. A gene acquires its being a gene in language. It is “languaged” as a gene. One need not speak the word “gene” in order to articulate “gene-ness”; to sequence or to clone is an act of languaging, reiterating the interpretation of the thing as a gene. Reality began to include genes when human interpretation disclosed “gene” as a possible meaning. 

The basic operation that humans perform in life is the operation of distinction; “whatever takes place in the praxis of living of the observer takes place as distinctions in language through languaging, and this is all that he or she can do as such” (16). Distinctions, the backbone of sense making, live in language; they are the access to what we don’t know we don’t know. Sensemaking “takes place in interactive talk and draws on the resources of language in order to formulate and exchange through talk…. As this occurs, a situation is talked into existence and the basis is laid for action to deal with it” (17). Bruffee (18) further explains:

We do not generate knowledge … by ‘dealing with’ the physical reality that shoves us around. We generate knowledge by ‘dealing with’ our beliefs about the physical reality that shoves us around. Specifically, we generate knowledge by justifying those beliefs socially.

Sensemaking, i.e., making something “sensible,” is a uniquely human capability (19). It is an iterative process of continuously co-authoring an evolving story so that it becomes more comprehensive, more comprehensible, and more durable in the face of challenge or doubt. Before we understand events, they are just occurrings; once we make sense of them they become explanations, knowledge, and reality. Richard Feynman’s (Nobel laureate, Physics, 1965) constant questioning of others’ findings meant that he had to recreate for himself much of what had been discovered by colleagues until it was clear for him. Written on Feynman’s blackboard at the time of his death was the following phrase, “What I cannot create, I do not understand” (20). 

## Sense-Making, Language, and Discovery

Science is the method most commonly used to discover knowledge and acquire an understanding of world we live in (the word “science” draws from the Latin scientia -- to know). It is useful to distinguish between discovering (a verb) and discovery (a noun). The term “discovering” refers to a process, generally the act of conducting research in the laboratory, the clinic, or the field, with the goal of uncovering a new finding or making a discovery. To designate a new finding, a “discovery” requires social certification. In general, a scientific paper whose results are largely accepted by the relevant scientific community is a discovery. Gross (21) elaborates:

A scientific discovery is the public attribution of novelty to a claim regarded by the relevant scientific community as possible and as the consequence of following appropriate methods. These criteria … behave just like conditions that are individually necessary and jointly sufficient for the attribution of scientific discovery.

Noe (22) explains that “the very term ‘discovery’ or ‘to discover’ etymologically stemmed from ‘to get rid of a cover.’ A discovery arises when something wearing a veil becomes explicit for us through removing obstacles. Generally speaking, it can be defined as the act of becoming aware of something previously existing but unknown.” Brannigan’s contention that “discoveries are social events whose statuses as discoveries are retrospectively and prospectively objectified” (23) is consistent with the theory of social construction, which assumes that there is no such thing as a universal foundation of knowledge. There is only agreement, a consensus arrived at for the time being by communities of knowledgeable peers (18). For example, Isaac Newton dominated the history of science not so much because he discovered the laws of motion that bear his name, but because he found an enduring and publicly accepted way of talking about the subject (24). Thus, theories, reality, and facts are language constructs generated by knowledge communities and used by them to maintain meaning through a shared conversational domain.

Scientific truth requires “unconcealment”, often by means of some form of measurement; “what is available for human use, becomes meaningful only by becoming visible as a thing of a particular kind…. [T]ruth is also a consequence of sight – we bring things to light, we unconceal them” (25). Language, however, is not the sole vehicle for sense-making. Interpretation can exist in the absence of words; “interpretation is carried out primordially … in an action of circumspective concern … without wasting words (26). Furthermore, language is not a replacement for intuition, the “gut feeling” or hunch that occurs without apparent reasoning, inference, or explanation. Gross emphasizes the importance of both the verbal and the visual in the discovery-dissemination continuum and their interrelatedness in the shift “from seeing to seeing as, from sight to insight.” He writes:

Although scientific visuals reveal the causal structure of the world, without language, that is, without the concepts that language embodies, what is revealed can neither be fully understood nor can this understanding be conveyed to others. In science, therefore, the visual can never be sufficient…. The verbal is necessary for this process: a visual cannot tell us in what ways it represents; only language can perform these functions (25).

Thus, while there exists a “poverty of language as a medium for conveying accurate, as opposed to evocative, descriptions”(27), we always stand within language even when we try to talk about phenomena that are difficult to describe. Human beings cannot perceive or interpret the world other than in the terms that they use to do so. We can never step outside of language to obtain a more accurate view of the world because there is no world for humans outside of language. In Gadamer’s words: “[N]ot only is the world ‘world’ only insofar as it comes into language, but language … has no independent life apart from the world that comes to language within it” (28). When we try to understand what’s outside of language we have to use language to do so. Thus, while there are things in our consciousness that surely exist independent of language, the only way we can talk about them – however ill-defined they may be – is with language.

Alfred Korzybski(29) coined the phrase, “the map is not the territory”, which captures his assessment that an abstraction of some thing is not the thing itself. This metaphor points to the impossibility of knowing what the territory actually (objectively) is, as any comprehension of it is based on some interpretive representation. In other words, maps are human constructions and they always reflect the mapmaker’s bias. Bateson (30) explains:

We say the map is different from the territory. But what is the territory? Operationally, somebody went out with a retina or a measuring stick and made representations which were then put on paper. What is on the paper map is a representation of what was in the retinal representation of the man who made the map; and as you push the question back, what you find is an infinite regress, an infinite series of maps. The territory never gets in at all…. Always, the process of representation will filter it out so that the mental world is only maps of maps, ad infinitum.

Sense-making is at the core of discovery. The discovery process involves observers drawing on prior experiences, wrestling with discrepancies, and making new distinctions. “The experience of language,” emphasizes Edie (31), “is the experience of meaning par excellence; it is our route of access to the realm of ‘the meant,’ of ‘sense’ and ‘signification.’” For example, whatever mental processes led Einstein to conclude that time was not constant, his inner thoughts, insights, and feelings at the moment were fashioned, at least in part, in language. Moreover, his discoveries could only be shared and made meaningful in language. This notion was stressed byGeorge Herbert Mead when he said (32): “Aperson who is saying something is saying to himself what he says to others; otherwise he does not know what he is talking about.”

The language of discovery provides researchers with a framework that enables them to experience the world of science and connect with colleagues uniquely. Sensemaking is a deliberate effort to understand events and concepts that we do not comprehend. Boje and colleagues (33) point out that “what we create in language ‘uses us’ in that it provides a point of view … within which we know reality and orient our actions.” In other words, the conversational domain of discovery, when mastered, become a context (a lens) that uses researchers to create the being, thinking, and action necessary to be experts in discovery.

Measurement is essential in science but measurement is not some thing that exists beforehand in the world that we later come to name; “how anything is studied in any science depends first upon the nature of humans as open to access[ing] . . . whatever is studied. Mathematics is not just there, its units and series have to be constituted by [human beings]. Physics isn't just there, human observation and measurement are certain specific modes of how humans are asgenerating time and space and things” (34). Computations and quantifications are human inventions that we overlay on our observations to give them meaning. Minutes, hours, and days are events of measurement, abstractly considered, that we are able to quantify; we partition the “occurringness” of the world into time by talking about its temporality. Deetz (4) explains: “That which is revealed, understood, and held is in language…. Things without words are static entities; language makes things into possibilities of experience…. The object is constituted – given its specific nature – only in the human encounter.” Language “coins” concepts so they are “shareable” beyond the context in which they were created, making them available to other conversational arenas. Without language, what is a photon? How long is human gestation? How much is pi?

When we observe someone being a scientist or “conducting science,” we see that person operating in the sphere of language. And, when you and I are being scientists we are operating in the sphere of language. Caneva (35) explains: “Perhaps the most important point about the characterization of any discovery is that, in order to be intelligible, it must be phrased in language understood by the intended audience, in language that typically implicates the taken-for-granted reality of that audience.”

Take cellular respiration or insulin secretion. Are they really things or are they actually complex events that we can treat as if they are things? Consider a surgical procedure, such as a colectomy. The surgeon opens the abdomen, mobilizes the colon, divides the bowel, ligates the vessels, does the anastamosis, repairs the mesenteric defect, and closes the abdomen. There is a continuous state of movement between hands and surgical instruments as if it’s one continuous happening, yet we can count the number of clamps, ties, needles, and minutes. All these things that exist in the world of surgery exist because we’re able to transform events into objects. We can only quantify objects if we detach them from their embededness in the experiential observed world. The world that humans live in is a meaningful world of objects that have no meaning apart from us. In Dewey’s words, “When communication occurs … events turn into objects, things with a meaning” (36).

To discover – to create meaning from questions that do not (yet) have answers – we must often change the context inside of which those questions are posed. Context can be defined as a set of hidden and unchallenged assumptions that color and shape the way in which the world “shows up for us and happens” (37). A contextual framework is a way of “seeing” in such a way that it provides a shared language that can be used by practitioners (and uses them) to communicate, perform, and innovate. Van Hecke (38) expounds: “People who win the Nobel Prize do so not because their work involves a high level of abstraction but because they overcame blind spots. They saw possibilities others rejected out of hand or grasped a perspective no one else had considered.”

Rather than considering language exclusively as a communication vehicle, scientists must also ask themselves how the findings they are dealing with become contextually meaningful. Language is always implicitly indexed to a prior recurring context(s) of reference; our current scientific understandings will undoubtedly seem limited if not primitive to those living a century from now (39,40). The earth became round, the solar system became heliocentric, and the universe became infinite when human thinking distinguished those possibilities. Einstein (41) offers a useful metaphor:

In our endeavor to understand reality, we are somewhat like a man trying to understand the mechanism of a closed watch. He sees the face and the moving hands, even hears it’s ticking, but he has no way of opening the case. If he is ingenious, he may form some picture of a mechanism which could be responsible for all the things he observes, but he will never be quite sure his picture is the only one which could explain his observations. He will never be able to compare his pictures with the real mechanism, and he cannot even imagine the possibility or the meaning of such a comparison.

Ingenuity and imagination fuel new distinctions; in distinguishing we perceive something previously unknown or unnoticed, which is called into being with appropriate language. A scientific breakthrough often depends on a critical, unexpected insight such that ways of thinking are used in one realm, are applied, as no one else has considered, in a different realm (42). In other words, “to know something that was not known before, one must be able to imagine it. If we speculate that there was a time when there was no knowledge, then the first act was not an act of knowing, but an act of imagination. The engine of all human knowing is not facts, or even experience. It is imagination.” (43).  

Pasteur reminds us that “in the field of observation, chance favors only the prepared mind” (44). Such preparation requires contextual intelligence - an intuitive grasp of relevant past events, an acute awareness of present contextual variables, and awareness of the preferred future (45). Because organizational realities are negotiated products, chance also favors the connected mind (46). “When people engage in acts of sensemaking,” Weick asserts, “it is more precise to think of them as accomplishing reality rather than discovering it” (19). Good ideas come from cultivated fluid networks of idea-sharing; “what is generally accepted as scientific knowledge is essentially the outcome of a process by which knowledge is reshaped as it passes through the hands of people with different agendas using different language” (47). 

## Our Distinctions Define the Limits of What We See as Possible

It is an implicit assumption of the scientific method that there is a right answer to the questions posed by science. But answers are only as good as the language within which they and the questions that generate them are framed. Scientific formulations are human constructions; thus, the intrinsic assumptions and frames of reference that scientists inescapably bring with them will always prevent the scientific process from being perfect. Gusfield (48) writes:

Description implies differentiation. The concepts we use require contrast. Nothing, except God, can exist without a context. To define, to “split,” necessitates a negation; saying what the object is not in order to say what it is. To define we must distinguish; we must divide the object from its context, indicating what it is by what it is not.

Thus, whatever takes place in the world of the scientist takes place as distinctions. Seeing new distinctions opens up possibilities for thinking and behaving in different ways. In the operation of distinction scientists bring forth content as well as the context in which it is distinguished. Contexts are constructed in language - there are no meanings that are context-free because conversations are always tied to prior contexts. The researcher who is adept with techniques and assays but is not adept with language (and hence is limited in his ability to create distinctions) is not a great scientist. In the absence of new distinctions we live predictably rather than creatively. 

Kenneth Burke introduced the expression “terministic screen ,” a set of symbols that becomes a kind of grid of intelligibility through which we make sense of the world; “the terms or vocabulary we use as a result of our occupations constitute a kind of screen that directs our attention to particular aspects of reality rather than others (49).” Language, Burke argued, doesn't simply “reflect” reality; it also helps select reality as well as deflect reality. Our “situatedness” prevents us from directly accessing the real world or having true knowledge about it. This is not to say that the objective world is not there, only that we can never shed our perspectives to access it. No one has a “god’s eye view” of reality; therefore no one can claim to have the truth about it. Stan Fish (50) describes this dilemma as follows:

Not only is there no one who could spot a transcendent truth if it happened to pass through the neighborhood, but it is difficult even to say what one would be like. Of course we would know what it would not be like; it would not speak to any particular condition, or be identified with any historical production, or be formulated in the terms of any national, ethnic, racial, economic, or class traditions.

Just as Newton revealed the laws of motion, Copernicus disclosed the planetary relationships in our solar system. And just as Newton invented a vocabulary that made the phenomenon we know as gravity accessible in news ways, Copernicus invented the new language of heliocentricity. In doing so, he expanded our world: a Copernican solar system is not simply the old system with new labels. New possibilities for human interaction were born out of and brought forth by the new language. Bineham (51) explains:

Truth no longer denotes a subject’s rational certainty that thought conforms to objective reality; instead, truth amounts to what can be argumentatively validated by the community of interpreters who act within a hermeneutic medium. Truth becomes a matter of what the medium will allow and what one’s interlocuters will accept. But the primary component of truth and reality remains the arguments and good reasons one can offer in support of a particular contention.

For illustrative purposes, it is useful to stress this concept more assertively. For example, the self-absorbed, arrogant king of England, Henry VIII (reign, 1509-1547),was not a narcissist; narcissism was invented by Havelock Ellis (52) in 1898 and wasn’t available duringHenry VIII's lifetime. This notion is counterintuitive to most people asthey debate that narcissism, as a human trait, has always beenthere. But narcissism is just a word created by Ellis as a way of clarifyinghis observations of human behavior. The utility of Ellis’distinction is not that he discovered something about human naturebut ratherthat he created a new language. Similarly, when Pasteur “languaged” the germ theory of disease, refuting the “spontaneous generation,” a new linguistic realm was created, one that (over time) came to include terms such as microbes, infection, and immunity. In acquiring meaning, what was discovered (microbiology) became accessible and served as a spark for the invention of pasteurization, antisepsis, and vaccines.

When a newborn looks into its mother’s face, what does it see? It neural capacity to perceive, differentiate, interpret, and reason is, arguably, greatly limited. It has no means by which to package, blend, and link together an infinite number of details – skin, eyes, nose, smile, hair, cheeks, forehead, lips - into a single entity known as mom. What the infant sees is a vast patchwork of constantly shifting colors and shapes. It has no words (yet) that make a color a color, a nose a nose, or a smile a smile. The babe has no linguistic context yet it is arguably discovering. Language is not essential for discovery (animals discover routinely) but it does augment hermeneutic complexity.

At the same time, language divides the inseparable whole into component parts, grants identity to each part, and names that part with a word. It creates our world, our identity, and our relationships. Words fragment, holding the rest of infinity at bay. “Things” in the world exist only because we have labeled them with words. Yet, language does not describe the world we see - we see the world language describes. As scientists who discover and create new knowledge, the distinctions we make in our thinking define the limits of what is possible. Distinctions jar us loose from our entrenched views such that we see an existing situation from a different vantage point or in a completely new light. 

## Using Language More Effectively

Knowledge is not something that exists independent of humans (53). There is much that is not known but there isn’t something out there in the world that is known that isn’t known by someone. Yet, we can never know some “thing” objectively, as it actually is. All we can do is observe it, interpret it, describe it, and quantify it. There is a prevailing conversational domain of discovery that has existed for centuries. It includes words and phrases like hypothesis testing, variability, sample size, study design, randomization, and statistical significance, terms that are familiar to all scientists. The terms and terminologies that make up this familiar conversational domain of discovery are intended to structure learning, access, and practice.

This prevailing language has resulted in enormous advances in science. But it often overlooks our human ways of being and acting that limit our effectiveness as researchers. For example, “the eye sees only what the mind is prepared to comprehend” and we often fail to recognize that our mental maps are our unique reality constructs (8). The way we believe the world “works” is often quite different than the way others think it works. Our beliefs are merely our perception of how the world works. Once our mental maps become etched, we often distort external inputs so they validate our views. This can lead to scientific bias. Until we decompress these constraints, we have little access to ways of thinking that lie outside the way we normally think. Becoming aware of these tendencies creates the possibility of loosening the confining grasp they have on us. 

 An evolving conversational domain increases our awareness of these ontological constraints (10,54). It requires mastery of new terms and terminologies, which include expressions such as already-always-listening, the occurring world, the way you wound up being, and our So-So future. For example, all human beings acquire early on an “already-always-listening” that filters and distorts virtually everything they encounter; scientists are no exception. This listening may show up for us as that ever-present voice that we are often unaware of. While we each have our own “listenings”, common ones include: “I know my research is better than his research”, “I’m probably going to get the shaft from Study Section”, and “I’m not good enough the way I am.” This latter listening is universal.

In order to cope with the listening that says we might fail or not measure up as scientists, we tend to default to certain ways of being and acting that we learned early on. They are solutions for dealing with our perceived inadequacies. By the time we are young adults, we have each incorporated a set of ways of being and acting that seem to give us a certain measure of success (8). Blaming, judging, and making excuses are common ways most of us “wound up being,” at least some of the time. If you decided as a child that you weren’t smart enough, your life-long “go-to” (automatic) strategy could be to overachieve in an attempt to convince yourself and others that you do measure up. Get more grants and publish more papers – whatever it takes to look smart.

Part of what gets in the way of being a superb scientist and performing superior science (discovery) is these limiting ways we wound up being. For example, if your listening is that others can’t be trusted, you will probably be reluctant to share your research findings and participate in team science. If as a scientist your default way of being under duress is to belittle your students, you are unlikely to “show up” as a good role model. If one’s range of possible ways of being and acting are limited, they will only be effective in situations that fit that range. Expanding one’s repertoire of behaviors will allow for competent leadership under a broader range of situations. But first we must become aware of our default ways of being.

The way we choose to speak to others and toourselves about our challenges shapesand colors the way they occur for us. For example, when we tell ourselves that the particular circumstances we are dealing with are “horrible” (e.g., my grant did not get funded), such an assessment only lives in language. “Horrible” is an interpretation (a context) we add to the details of the situation. We can fall into the trap of believing that the only future ahead of us is a So-So (same ’ol, same ’ol) future, one that is a largely continuation of the past. This So-So future provides the thinking construct from which we try to change our lives but nothing much happens. A conversational domain that gives researchers access to their worldviews and mental maps that limit their ways of thinking about science and doing science will enhance their performance.

The best scientists are aware that there is much that they don’t know they don’t know. This is the territory that offers the greatest opportunity for insights and learning. The greatest access to this domain comes from peers who provide us with feedback. Top scientists use language to recontextualize their approach to problem-solving, which elicits new insights that result in new discoveries. When prevailing contexts are linguistically unveiled, new contexts can be created that shift the way our research challenges occur for us. This provides scientists with new opportunity sets (previously unavailable) for breakthroughs. 

Transformative learning requires shifting an entrenched frame of reference, a process that involves critically self-reflecting on the assumptions upon which our interpretations, beliefs, and habits of mind are based (55,56). Such learning is not about changing one’s or another’s mind to adopt the “right” point of view but rather about becoming more receptive to new ways of thinking, perceiving, and acting differently in the world. The purpose of reflection is to become aware of one’s biases and assumptions - to bracket them or set them aside - in order to engage the experience without preconceived notions. This requires an attentiveness to the ways in which language is used and an awareness of life as an interpretive experience; to see something in a new imaginative way is to see it other than it has been seen before and to integrate it into a new linguistic context (57,58). This new thinking emerges out of new ontological distinctions at the level of what is unspoken. Anderson (59) stresses that the real realm of scientific inquiry is “not primarily the laboratory; much of the difficult work is performed first through [the] process of refining the language in which the question is asked.”

The products of science - discoveries, knowledge, new technologies - have given us astounding dominion over our planet. Yet, “a closer reading of Homo sapiens,” writes Orr (60), “would suggest that at best we are a spindly legged, upstart, disruptive species whose intellect exceeds its wisdom, located on a small planet attached to an insignificant star in a backwater galaxy.” The dangers of not being able to manage modernization are not esoteric or academic. “Technology” contends Herzogenrath (61), “somehow works and functions in and for itself, simultaneously producing man as subject and erasing the subject as an autonomous entity in control. Houston (62) amplifies:

Humankind has fallen far behind the advancements in technology. The precarious state of [global] imbalance that we are now experiencing is an obvious sign of the power of technology far exceeding the power of human beings to be in control of it. It could easily be argued that we have fallen far behind the advancements in technology, simply because the languages we use for daily communication do not help us to make the distinctions required to be in balance with the technology that has taken over our lives.

The imprecision of language is part of our struggle to understand the technological world we live in. Moreover, “the carving up of nature, its reduction into concepts and equivalences, occurs along lines laid down by the patterns of language.  And the more the machinery of language … subjects existence to itself, the more blind its role in reproducing a society of subjugation” (63). Schön (64) characterizes the critical inseparability of thinking and action:

When somebody reflects-in-action, he becomes a researcher in the practice context…. He does not keep means and ends separate, but defines them interactively as he frames a problematic situation. He does not separate thinking from doing, ratiocinating his way to a decision which he must later convert to action.”

However, reflection-in-action – what Schön refers to as “dialoguing with the material at hand” - is not a common method of learning; most professionals take action based on beliefs and theories, failing to appreciate the reality of exercising knowledge “in action”, as lived, in real situations. Yet, “in the prison-house of language,” writes Gusfield (48), “it is important to search for the key even if we never find it. The process is itself transformative.” Heidegger (65) calls for a way of living with technology that does not allow it to “warp, confuse, and lay waste our nature.” This new way of living can only be envisioned, accessed, and constituted in language. Gene Gendlin, 2008 winner of the Viktor Frankl Prize, elaborates (66):

As we look about us in the city today, we find ourselves surrounded by man-made things, by technologically determined routines and views. There has been a silencing of nature, including our own nature…. It misses being and may enslave us to what we have made…. [We] must reinterpret, newly interpret, invent meaning …, and generate new futures and new significances in order to mold the already given troubling meanings of [our] situation.

How do we rescue language? Maddocks (67) asks, “How are words repaired, put back in shape, restored to accuracy and eloquence, made faithful again to the commands of the mind and the heart? There is, sadly enough, no easy answer…. All of us—from the admen with their jingles to the tin-eared scholars with their jargon—are victims as well as victimizers of the language we have inherited.” Whether modern technology realizes its “supreme danger” or “saving power” resides in our ability to listen, reflect, and use transformative language (65). By virtue of its symbolizing power, “language creates false separations and objectifications.  This falsification is made possible by concealing, and ultimately vitiating, the participation of the subject in the physical world…. (63)” Thus, to use language is to restrict oneself to the modes of perception already inherent in that language. Distinguishing that the world is shaped by and accessible in language does not tell us how to talk or what to say, but it does provide us with the possibility of having a say in the future we create. “Modern society,” Ignatieff says, “is changing the locus of belonging…. We need justice, we need liberty, and we need as much solidarity as can be reconciled with justice and liberty. But we also need, as much as anything else, language adequate to the times we live in” (68). This language is not just about new concepts and terminologies; rather, it is a world of richer linguistic distinctions, one that begins “with the realization of the need to struggle with words to make them do more fully what we wish them to do (24).” 

Science is a journey that our species has been on for the past two and a half millennia in an attempt to come to grips with how the universe works and what it means to be human. Aristotle begins his masterpiece Metaphysics, written nearly 2400 years ago, by pointing out that all human beings are naturally curious because of their innate desire to know (69). Curiosity breeds inquiry, which leads to discovery and progress. Language is a key means by which the unknown is made meaningfully known and accessible for the benefit of science and humankind. Through language our collective sensemaking and sensegiving can create the wisdom to solve the world’s problems prudently and compassionately and, in so doing, contribute to global transformation. 
